# Supplementary material for: Evaluation of female Aedes aegypti proteome via LC-ESI-MS/MS using two protein extraction methods
Source: PeerJ. 2021 Mar 3;9:e10863. doi: 10.7717/peerj.10863 (PMC7936558; doi:10.7717/peerj.10863)
Supplement: Supplemental Information 1 — A list of 20 top proteins using LC-ESI-MS/MS [file peerj-09-10863-s001.docx]

**Supplementary file 1**

**Evaluation of female *Aedes aegypti* proteome *via* LC-ESI-MS/MS using two protein extraction methods**

Table 1: Top 20 proteins based on the highest -10lgp score TCA acetone precipitation extracted proteins replicate 1

| **SN** | **Accession** | **-10lgP** | **Coverage (%)** | **#Peptides** | **#Unique** | **Avg. Mass** | **Description** |
| --- | --- | --- | --- | --- | --- | --- | --- |
| 1. | tr\|Q179E8\|Q179E8_AEDAE | 238.89 | 33 | 68 | 15 | 221462 | AAEL005656-PA OS=Aedes aegypti OX=7159 GN=AAEL005656 PE=3 SV=1 |
| 2. | tr\|W0FUL2\|W0FUL2_AEDAE | 234.71 | 31 | 63 | 15 | 221323 | Myosin heavy chain OS=Aedes aegypti OX=7159 GN=myo-sex PE=2 SV=1 |
| 3. | tr\|A0A1Q3G4T4\|A0A1Q3G4T4_CULTA | 230.61 | 24 | 51 | 15 | 224263 | Putative myosin class i heavy chain OS=Culex tarsalis OX=7177 PE=3 SV=1 |
| 4. | tr\|Q178Y4\|Q178Y4_AEDAE | 229.19 | 24 | 50 | 15 | 224208 | AAEL005733-PB OS=Aedes aegypti OX=7159 GN=AAEL005733 PE=3 SV=1 |
| 5. | tr\|Q178Y3\|Q178Y3_AEDAE | 229.19 | 24 | 50 | 15 | 222221 | AAEL005733-PA OS=Aedes aegypti OX=7159 GN=AAEL005733 PE=3 SV=1 |
| 6. | tr\|B0W188\|B0W188_CULQU | 220.81 | 22 | 46 | 15 | 219420 | Myosin heavy chain OS=Culex quinquefasciatus OX=7176 GN=6031761 PE=3 SV=1 |
| 7. | tr\|A0A1S4H419\|A0A1S4H419_ANOGA | 220.7 | 21 | 44 | 15 | 224088 | Myosin heavy chain OS=Anopheles gambiae OX=7165 PE=3 SV=1 |
| 8. | tr\|A0A182XLF1\|A0A182XLF1_ANOQN | 220.7 | 18 | 44 | 15 | 253203 | Uncharacterized protein OS=Anopheles quadriannulatus OX=34691 PE=3 SV=1 |
| 9. | tr\|A0A182V4B9\|A0A182V4B9_ANOME | 220.37 | 19 | 44 | 15 | 246591 | Uncharacterized protein OS=Anopheles merus OX=30066 PE=3 SV=1 |
| 10. | tr\|A0A2M4CTP0\|A0A2M4CTP0_ANODA | 220.21 | 21 | 44 | 15 | 221726 | Putative myosin class i heavy chain OS=Anopheles darlingi OX=43151 PE=3 SV=1 |
| 11. | tr\|A0A182HQV8\|A0A182HQV8_ANOAR | 218.41 | 18 | 42 | 15 | 254328 | Uncharacterized protein OS=Anopheles arabiensis OX=7173 PE=3 SV=1 |
| 12. | tr\|A0A1S4H355\|A0A1S4H355_ANOGA | 218.41 | 20 | 42 | 15 | 223756 | Myosin heavy chain OS=Anopheles gambiae OX=7165 PE=3 SV=1 |
| 13. | tr\|A0A1Q3G4C2\|A0A1Q3G4C2_CULTA | 218.22 | 20 | 42 | 15 | 221303 | Putative myosin class i heavy chain OS=Culex tarsalis OX=7177 PE=3 SV=1 |
| 14. | tr\|A0A1Q3FI37\|A0A1Q3FI37_CULTA | 205.92 | 16 | 35 | 15 | 222981 | Putative myosin class i heavy chain OS=Culex tarsalis OX=7177 PE=3 SV=1 |
| 15. | tr\|Q6QNY2\|Q6QNY2_AEDAE | 186.74 | 42 | 26 | 7 | 41582 | AAEL001951-PA OS=Aedes aegypti OX=7159 GN=5572985 PE=2 SV=1 |
| 16. | tr\|A0A182H2T8\|A0A182H2T8_AEDAL | 186.74 | 42 | 26 | 7 | 41566 | Uncharacterized protein OS=Aedes albopictus OX=7160 GN=109412625 PE=3 SV=1 |
| 17. | tr\|B0WZI4\|B0WZI4_CULQU | 178.1 | 38 | 22 | 7 | 41598 | Actin-5 OS=Culex quinquefasciatus OX=7176 GN=6045432 PE=3 SV=1 |
| 18. | tr\|Q4JQ54\|Q4JQ54_CULPP | 176.89 | 38 | 22 | 7 | 41810 | Actin OS=Culex pipiens pipiens OX=38569 PE=2 SV=1 |
| 19. | tr\|A0A182GF96\|A0A182GF96_AEDAL | 176.89 | 38 | 22 | 7 | 41643 | Uncharacterized protein OS=Aedes albopictus OX=7160 GN=109412623 PE=3 SV=1 |
| 20. | tr\|A0A182XUG6\|A0A182XUG6_ANOQN | 176.89 | 38 | 22 | 6 | 41659 | Uncharacterized protein OS=Anopheles quadriannulatus OX=34691 PE=3 SV=1 |

Table 2: Top 20 proteins based on the highest -10lgp score TCA acetone precipitation extracted proteins replicate 2

| **SN** | **Accession** | **-10lgP** | **Coverage (%)** | **#Peptides** | **#Unique** | **Avg. Mass** | **Description** |
| --- | --- | --- | --- | --- | --- | --- | --- |
| 1. | tr\|A0A3F2YYT0\|A0A3F2YYT0_ANOGA | 133.71 | 37 | 16 | 16 | 48804 | ATP synthase subunit beta OS=Anopheles gambiae OX=7165 PE=3 SV=1 |
| 2. | tr\|A0A1S4H9B2\|A0A1S4H9B2_ANOGA | 133.71 | 34 | 16 | 16 | 53143 | ATP synthase subunit beta OS=Anopheles gambiae OX=7165 PE=3 SV=1 |
| 3. | tr\|A0A1S4F4H1\|A0A1S4F4H1_AEDAE | 133.71 | 34 | 16 | 16 | 53988 | ATP synthase subunit beta OS=Aedes aegypti OX=7159 GN=5577999 PE=3 SV=1 |
| 4. | tr\|A0A1S4H8F8\|A0A1S4H8F8_ANOGA | 133.71 | 34 | 16 | 16 | 54080 | ATP synthase subunit beta OS=Anopheles gambiae OX=7165 PE=3 SV=1 |
| 5. | tr\|A0A182X065\|A0A182X065_ANOQN | 133.71 | 34 | 16 | 16 | 54022 | ATP synthase subunit beta OS=Anopheles quadriannulatus OX=34691 PE=3 SV=1 |
| 6. | tr\|A0A1Q3FH39\|A0A1Q3FH39_CULTA | 133.71 | 34 | 16 | 16 | 53891 | ATP synthase subunit beta OS=Culex tarsalis OX=7177 PE=3 SV=1 |
| 7. | tr\|A0A182RSV0\|A0A182RSV0_ANOFN | 133.71 | 34 | 16 | 16 | 53974 | ATP synthase subunit beta OS=Anopheles funestus OX=62324 PE=3 SV=1 |
| 8. | tr\|A0A4Y0BGI4\|A0A4Y0BGI4_ANOFN | 133.71 | 34 | 16 | 16 | 53986 | ATP synthase subunit beta OS=Anopheles funestus OX=62324 PE=3 SV=1 |
| 9. | tr\|A0A182HPL3\|A0A182HPL3_ANOAR | 133.71 | 34 | 16 | 16 | 54022 | ATP synthase subunit beta OS=Anopheles arabiensis OX=7173 PE=3 SV=1 |
| 10. | tr\|Q17FL3\|Q17FL3_AEDAE | 133.71 | 34 | 16 | 16 | 53974 | ATP synthase subunit beta OS=Aedes aegypti OX=7159 GN=AAEL003393 PE=3 SV=1 |
| 11. | tr\|A0A023ETB9\|A0A023ETB9_AEDAL | 133.71 | 34 | 16 | 16 | 54017 | ATP synthase subunit beta OS=Aedes albopictus OX=7160 PE=2 SV=1 |
| 12. | tr\|Q17H12\|Q17H12_AEDAE | 133.71 | 34 | 16 | 16 | 53912 | ATP synthase subunit beta OS=Aedes aegypti OX=7159 GN=5576214 PE=3 SV=1 |
| 13. | tr\|A0A182RAH9\|A0A182RAH9_ANOFN | 133.71 | 34 | 16 | 16 | 54018 | ATP synthase subunit beta OS=Anopheles funestus OX=62324 PE=3 SV=1 |
| 14. | tr\|A0A023EUC8\|A0A023EUC8_AEDAL | 133.71 | 34 | 16 | 16 | 53912 | ATP synthase subunit beta OS=Aedes albopictus OX=7160 PE=2 SV=1 |
| 15. | tr\|E3XEC7\|E3XEC7_ANODA | 133.71 | 34 | 16 | 16 | 53768 | ATP synthase subunit beta OS=Anopheles darlingi OX=43151 GN=AND_006288 PE=3 SV=1 |
| 16. | tr\|A0A182FTY5\|A0A182FTY5_ANOAL | 133.71 | 34 | 16 | 16 | 53774 | ATP synthase subunit beta OS=Anopheles albimanus OX=7167 PE=3 SV=1 |
| 17. | tr\|T1DES1\|T1DES1_ANOAQ | 133.71 | 34 | 16 | 16 | 53790 | ATP synthase subunit beta OS=Anopheles aquasalis OX=42839 PE=2 SV=1 |
| 18. | tr\|A0A182FTY6\|A0A182FTY6_ANOAL | 133.71 | 34 | 16 | 16 | 53768 | ATP synthase subunit beta OS=Anopheles albimanus OX=7167 PE=3 SV=1 |
| 19. | tr\|A0A182UML8\|A0A182UML8_ANOME | 133.71 | 34 | 16 | 16 | 53727 | ATP synthase subunit beta OS=Anopheles merus OX=30066 PE=3 SV=1 |
| 20. | tr\|A0A182X083\|A0A182X083_ANOQN | 133.71 | 34 | 16 | 16 | 53727 | ATP synthase subunit beta OS=Anopheles quadriannulatus OX=34691 PE=3 SV=1 |

Table 3: Top 20 proteins based on the highest -10lgp score TCA acetone precipitation extracted proteins replicate 3

| **SN** | **Accession** | **-10lgP** | **Coverage (%)** | **#Peptides** | **#Unique** | **Avg. Mass** | **Description** |
| --- | --- | --- | --- | --- | --- | --- | --- |
| 1. | tr\|Q1HRQ7\|Q1HRQ7_AEDAE | 141.29 | 23 | 15 | 15 | 59392 | ATP synthase subunit alpha OS=Aedes aegypti OX=7159 GN=5575914 PE=2 SV=1 |
| 2. | tr\|A0A023EUC8\|A0A023EUC8_AEDAL | 141.27 | 25 | 13 | 13 | 53912 | ATP synthase subunit beta OS=Aedes albopictus OX=7160 PE=2 SV=1 |
| 3. | tr\|A0A3F2YYT0\|A0A3F2YYT0_ANOGA | 141.27 | 28 | 13 | 13 | 48804 | ATP synthase subunit beta OS=Anopheles gambiae OX=7165 PE=3 SV=1 |
| 4. | tr\|Q1HR61\|Q1HR61_AEDAE | 141.27 | 25 | 13 | 13 | 53971 | ATP synthase subunit beta OS=Aedes aegypti OX=7159 PE=2 SV=1 |
| 5. | tr\|B0WGW0\|B0WGW0_CULQU | 141.27 | 25 | 13 | 13 | 54589 | ATP synthase subunit beta OS=Culex quinquefasciatus OX=7176 GN=6038107 PE=3 SV=1 |
| 6. | tr\|A0A182RAH9\|A0A182RAH9_ANOFN | 141.27 | 25 | 13 | 13 | 54018 | ATP synthase subunit beta OS=Anopheles funestus OX=62324 PE=3 SV=1 |
| 7. | tr\|A0A023ETB9\|A0A023ETB9_AEDAL | 141.27 | 25 | 13 | 13 | 54017 | ATP synthase subunit beta OS=Aedes albopictus OX=7160 PE=2 SV=1 |
| 8. | tr\|Q17FL3\|Q17FL3_AEDAE | 141.27 | 25 | 13 | 13 | 53974 | ATP synthase subunit beta OS=Aedes aegypti OX=7159 GN=AAEL003393 PE=3 SV=1 |
| 9. | tr\|A0A4Y0BGI4\|A0A4Y0BGI4_ANOFN | 141.27 | 25 | 13 | 13 | 53986 | ATP synthase subunit beta OS=Anopheles funestus OX=62324 PE=3 SV=1 |
| 10. | tr\|A0A182RSV0\|A0A182RSV0_ANOFN | 141.27 | 25 | 13 | 13 | 53974 | ATP synthase subunit beta OS=Anopheles funestus OX=62324 PE=3 SV=1 |
| 11. | tr\|A0A1Q3FH39\|A0A1Q3FH39_CULTA | 141.27 | 25 | 13 | 13 | 53891 | ATP synthase subunit beta OS=Culex tarsalis OX=7177 PE=3 SV=1 |
| 12. | tr\|A0A182X065\|A0A182X065_ANOQN | 141.27 | 25 | 13 | 13 | 54022 | ATP synthase subunit beta OS=Anopheles quadriannulatus OX=34691 PE=3 SV=1 |
| 13. | tr\|A0A1S4F4H1\|A0A1S4F4H1_AEDAE | 141.27 | 25 | 13 | 13 | 53988 | ATP synthase subunit beta OS=Aedes aegypti OX=7159 GN=5577999 PE=3 SV=1 |
| 14. | tr\|A0A182FTY5\|A0A182FTY5_ANOAL | 141.27 | 25 | 13 | 13 | 53774 | ATP synthase subunit beta OS=Anopheles albimanus OX=7167 PE=3 SV=1 |
| 15. | tr\|E3XEC7\|E3XEC7_ANODA | 141.27 | 25 | 13 | 13 | 53768 | ATP synthase subunit beta OS=Anopheles darlingi OX=43151 GN=AND_006288 PE=3 SV=1 |
| 16. | tr\|A0A182X083\|A0A182X083_ANOQN | 141.27 | 25 | 13 | 13 | 53727 | ATP synthase subunit beta OS=Anopheles quadriannulatus OX=34691 PE=3 SV=1 |
| 17. | tr\|A0A182UML8\|A0A182UML8_ANOME | 141.27 | 25 | 13 | 13 | 53727 | ATP synthase subunit beta OS=Anopheles merus OX=30066 PE=3 SV=1 |
| 18. | tr\|A0A1S4H9B2\|A0A1S4H9B2_ANOGA | 141.27 | 26 | 13 | 13 | 53143 | ATP synthase subunit beta OS=Anopheles gambiae OX=7165 PE=3 SV=1 |
| 19. | tr\|A0A084WIY6\|A0A084WIY6_ANOSI | 141.27 | 25 | 13 | 13 | 54048 | ATP synthase subunit beta OS=Anopheles sinensis OX=74873 GN=ZHAS_00018244 PE=3 SV=1 |
| 20. | tr\|A0A1S4FIY1\|A0A1S4FIY1_AEDAE | 94.37 | 19 | 8 | 8 | 35296 | Uncharacterized protein OS=Aedes aegypti OX=7159 GN=5570233 PE=3 SV=1 |

Table 4: Top 20 proteins based on the highest -10lgp score Cytobuster extracted proteins replicate 1

| **SN** | **Accession** | **-10lgP** | **Coverage (%)** | **#Peptides** | **#Unique** | **Avg. Mass** | **Description** |
| --- | --- | --- | --- | --- | --- | --- | --- |
| 1. | tr\|Q1HR61\|Q1HR61_AEDAE | 202.23 | 58 | 35 | 27 | 53971 | ATP synthase subunit beta OS=Aedes aegypti OX=7159 PE=2 SV=1 |
| 2. | tr\|A0A182FTY5\|A0A182FTY5_ANOAL | 202.23 | 58 | 35 | 27 | 53774 | ATP synthase subunit beta OS=Anopheles albimanus OX=7167 PE=3 SV=1 |
| 3. | tr\|A0A1S4F4H1\|A0A1S4F4H1_AEDAE | 202.23 | 58 | 35 | 27 | 53988 | ATP synthase subunit beta OS=Aedes aegypti OX=7159 GN=5577999 PE=3 SV=1 |
| 4. | tr\|A0A182X065\|A0A182X065_ANOQN | 202.23 | 58 | 35 | 27 | 54022 | ATP synthase subunit beta OS=Anopheles quadriannulatus OX=34691 PE=3 SV=1 |
| 5. | tr\|A0A4Y0BGI4\|A0A4Y0BGI4_ANOFN | 202.23 | 58 | 35 | 27 | 53986 | ATP synthase subunit beta OS=Anopheles funestus OX=62324 PE=3 SV=1 |
| 6. | tr\|Q17FL3\|Q17FL3_AEDAE | 202.23 | 58 | 35 | 27 | 53974 | ATP synthase subunit beta OS=Aedes aegypti OX=7159 GN=AAEL003393 PE=3 SV=1 |
| 7. | tr\|A0A023ETB9\|A0A023ETB9_AEDAL | 202.23 | 58 | 35 | 27 | 54017 | ATP synthase subunit beta OS=Aedes albopictus OX=7160 PE=2 SV=1 |
| 8. | tr\|A0A023EUC8\|A0A023EUC8_AEDAL | 202.23 | 58 | 35 | 27 | 53912 | ATP synthase subunit beta OS=Aedes albopictus OX=7160 PE=2 SV=1 |
| 9. | tr\|A0A3F2YYT0\|A0A3F2YYT0_ANOGA | 202.23 | 64 | 35 | 27 | 48804 | ATP synthase subunit beta OS=Anopheles gambiae OX=7165 PE=3 SV=1 |
| 10. | tr\|Q16KR4\|Q16KR4_AEDAE | 173.56 | 27 | 21 | 21 | 85724 | Aconitate hydratase mitochondrial OS=Aedes aegypti OX=7159 GN=5580315 PE=3 SV=1 |
| 11. | tr\|Q17EL3\|Q17EL3_AEDAE | 173.56 | 27 | 21 | 21 | 87334 | Aconitate hydratase mitochondrial OS=Aedes aegypti OX=7159 GN=AAEL003734 PE=3 SV=1 |
| 12. | tr\|Q172T4\|Q172T4_AEDAE | 170.17 | 20 | 19 | 19 | 103646 | AAEL007306-PA OS=Aedes aegypti OX=7159 GN=AAEL007306 PE=4 SV=1 |
| 13. | tr\|A0A023EWA2\|A0A023EWA2_AEDAL | 170.17 | 20 | 19 | 19 | 103615 | Putative ca2+-binding actin-bundling protein OS=Aedes albopictus OX=7160 PE=2 SV=1 |
| 14. | tr\|Q178U9\|Q178U9_AEDAE | 174.83 | 47 | 18 | 18 | 39120 | Fructose-bisphosphate aldolase OS=Aedes aegypti OX=7159 GN=5567031 PE=3 SV=1 |
| 15. | tr\|A0A023EQM6\|A0A023EQM6_AEDAL | 174.83 | 47 | 18 | 18 | 39152 | Fructose-bisphosphate aldolase OS=Aedes albopictus OX=7160 PE=2 SV=1 |
| 16. | Q16P20\|CISY2_AEDAE | 167.65 | 23 | 15 | 15 | 51641 | Probable citrate synthase 2 mitochondrial OS=Aedes aegypti OX=7159 GN=AAEL011789 PE=3 SV=1 |
| 17. | Q17GM7\|CISY1_AEDAE | 167.65 | 23 | 15 | 15 | 51657 | Probable citrate synthase 1 mitochondrial OS=Aedes aegypti OX=7159 GN=AAEL002956 PE=3 SV=1 |
| 18. | tr\|A0A023ESP1\|A0A023ESP1_AEDAL | 167.65 | 23 | 15 | 15 | 51576 | Citrate synthase OS=Aedes albopictus OX=7160 PE=2 SV=1 |
| 19. | tr\|T1E2L3\|T1E2L3_9DIPT | 167.65 | 23 | 15 | 15 | 51646 | Citrate synthase OS=Psorophora albipes OX=869069 PE=2 SV=1 |
| 20. | tr\|A0A182GKM3\|A0A182GKM3_AEDAL | 167.65 | 23 | 15 | 15 | 51502 | Citrate synthase OS=Aedes albopictus OX=7160 GN=RP20_CCG011547 PE=3 SV=1 |

Table 5: Top 20 proteins based on the highest -10lgp Cytobuster extracted proteins replicate 2

| **SN** | **Accession** | **-10lgP** | **Coverage (%)** | **#Peptides** | **#Unique** | **Avg. Mass** | **Description** |
| --- | --- | --- | --- | --- | --- | --- | --- |
| 1. | tr\|Q1HRQ7\|Q1HRQ7_AEDAE | 193.31 | 39 | 25 | 21 | 59392 | ATP synthase subunit alpha OS=Aedes aegypti OX=7159 GN=5575914 PE=2 SV=1 |
| 2. | tr\|A0A023EWA2\|A0A023EWA2_AEDAL | 179.43 | 23 | 20 | 20 | 103615 | Putative ca2+-binding actin-bundling protein OS=Aedes albopictus OX=7160 PE=2 SV=1 |
| 3. | tr\|Q172T4\|Q172T4_AEDAE | 179.43 | 23 | 20 | 20 | 103646 | AAEL007306-PA OS=Aedes aegypti OX=7159 GN=AAEL007306 PE=4 SV=1 |
| 4. | tr\|T1E2L3\|T1E2L3_9DIPT | 162.66 | 27 | 17 | 17 | 51646 | Citrate synthase OS=Psorophora albipes OX=869069 PE=2 SV=1 |
| 5. | tr\|A0A023ESP1\|A0A023ESP1_AEDAL | 162.66 | 27 | 17 | 17 | 51576 | Citrate synthase OS=Aedes albopictus OX=7160 PE=2 SV=1 |
| 6. | tr\|A0A182GKM3\|A0A182GKM3_AEDAL | 162.66 | 27 | 17 | 17 | 51502 | Citrate synthase OS=Aedes albopictus OX=7160 GN=RP20_CCG011547 PE=3 SV=1 |
| 7. | tr\|A0A1S4EZ75\|A0A1S4EZ75_AEDAE | 148.94 | 29 | 13 | 13 | 40568 | Glycerol-3-phosphate dehydrogenase [NAD(+)] OS=Aedes aegypti OX=7159 GN=5571293 PE=3 SV=1 |
| 8. | tr\|Q16F38\|Q16F38_AEDAE | 139.4 | 24 | 11 | 11 | 57457 | Pyruvate kinase OS=Aedes aegypti OX=7159 GN=5565629 PE=1 SV=1 |
| 9. | tr\|Q16LP5\|Q16LP5_AEDAE | 139.4 | 24 | 11 | 11 | 56218 | Pyruvate kinase OS=Aedes aegypti OX=7159 GN=AAEL012576 PE=3 SV=1 |
| 10. | tr\|Q17E81\|Q17E81_AEDAE | 131.57 | 17 | 11 | 11 | 80970 | Glycerol-3-phosphate dehydrogenase OS=Aedes aegypti OX=7159 GN=5579187 PE=3 SV=1 |
| 11. | tr\|Q17E82\|Q17E82_AEDAE | 131.57 | 17 | 11 | 11 | 81269 | Glycerol-3-phosphate dehydrogenase OS=Aedes aegypti OX=7159 GN=5579187 PE=3 SV=1 |
| 12. | tr\|Q17AK0\|Q17AK0_AEDAE | 139.81 | 23 | 10 | 10 | 45570 | AAEL005269-PA OS=Aedes aegypti OX=7159 GN=AAEL005269 PE=4 SV=1 |
| 13. | tr\|A0A1S4EXR8\|A0A1S4EXR8_AEDAE | 96.36 | 18 | 10 | 10 | 68763 | Malic enzyme OS=Aedes aegypti OX=7159 PE=3 SV=1 |
| 14. | tr\|Q17M99\|Q17M99_AEDAE | 96.36 | 17 | 10 | 10 | 72027 | Malic enzyme OS=Aedes aegypti OX=7159 GN=AAEL001091 PE=3 SV=1 |
| 15. | tr\|Q1HR67\|Q1HR67_AEDAE | 206.18 | 60 | 25 | 9 | 39874 | AAEL009185-PA OS=Aedes aegypti OX=7159 GN=5571596 PE=2 SV=1 |
| 16. | tr\|Q16XK3\|Q16XK3_AEDAE | 132.83 | 29 | 9 | 9 | 32751 | ATP synthase subunit gamma OS=Aedes aegypti OX=7159 GN=5571150 PE=3 SV=1 |
| 17. | tr\|Q17A27\|Q17A27_AEDAE | 126.8 | 21 | 9 | 9 | 63275 | Multifunctional fusion protein OS=Aedes aegypti OX=7159 GN=5566485 PE=3 SV=1 |
| 18. | tr\|A0A023ETA6\|A0A023ETA6_AEDAL | 121.08 | 27 | 9 | 9 | 46553 | Putative enolase OS=Aedes albopictus OX=7160 PE=2 SV=1 |
| 19. | tr\|Q17KK5\|Q17KK5_AEDAE | 121.08 | 27 | 9 | 9 | 46621 | AAEL001668-PA OS=Aedes aegypti OX=7159 GN=AAEL001668 PE=3 SV=1 |
| 20. | tr\|Q16KR4\|Q16KR4_AEDAE | 168.93 | 24 | 21 | 8 | 85724 | Aconitate hydratase mitochondrial OS=Aedes aegypti OX=7159 GN=5580315 PE=3 SV=1 |

Table 6: Top 20 proteins based on the highest -10lgp score Cytobuster extracted proteins replicate 3

| **SN** | **Accession** | **-10lgP** | **Coverage (%)** | **#Peptides** | **#Unique** | **Avg. Mass** | **Description** |
| --- | --- | --- | --- | --- | --- | --- | --- |
| 1. | tr\|Q17EL3\|Q17EL3_AEDAE | 172.8 | 33 | 25 | 25 | 87334 | Aconitate hydratase mitochondrial OS=Aedes aegypti OX=7159 GN=AAEL003734 PE=3 SV=1 |
| 2. | tr\|Q16KR4\|Q16KR4_AEDAE | 172.8 | 33 | 25 | 25 | 85724 | Aconitate hydratase mitochondrial OS=Aedes aegypti OX=7159 GN=5580315 PE=3 SV=1 |
| 3. | tr\|Q1HR67\|Q1HR67_AEDAE | 184.28 | 52 | 22 | 22 | 39874 | AAEL009185-PA OS=Aedes aegypti OX=7159 GN=5571596 PE=2 SV=1 |
| 4. | tr\|Q1HRQ7\|Q1HRQ7_AEDAE | 175.15 | 41 | 25 | 22 | 59392 | ATP synthase subunit alpha OS=Aedes aegypti OX=7159 GN=5575914 PE=2 SV=1 |
| 5. | tr\|Q172T4\|Q172T4_AEDAE | 160.59 | 20 | 19 | 19 | 103646 | AAEL007306-PA OS=Aedes aegypti OX=7159 GN=AAEL007306 PE=4 SV=1 |
| 6. | tr\|A0A023EWA2\|A0A023EWA2_AEDAL | 160.59 | 20 | 19 | 19 | 103615 | Putative ca2+-binding actin-bundling protein OS=Aedes albopictus OX=7160 PE=2 SV=1 |
| 7. | tr\|Q17KS3\|Q17KS3_AEDAE | 141.91 | 33 | 13 | 13 | 38178 | Glycerol-3-phosphate dehydrogenase [NAD(+)] OS=Aedes aegypti OX=7159 GN=AAEL001593 PE=3 SV=1 |
| 8. | tr\|Q17KS5\|Q17KS5_AEDAE | 141.91 | 32 | 13 | 13 | 38903 | Glycerol-3-phosphate dehydrogenase [NAD(+)] OS=Aedes aegypti OX=7159 GN=5571293 PE=3 SV=1 |
| 9. | tr\|Q17KS4\|Q17KS4_AEDAE | 141.91 | 32 | 13 | 13 | 39292 | Glycerol-3-phosphate dehydrogenase [NAD(+)] OS=Aedes aegypti OX=7159 GN=5571293 PE=3 SV=1 |
| 10. | tr\|A0A1S4EZ75\|A0A1S4EZ75_AEDAE | 141.91 | 31 | 13 | 13 | 40568 | Glycerol-3-phosphate dehydrogenase [NAD(+)] OS=Aedes aegypti OX=7159 GN=5571293 PE=3 SV=1 |
| 11. | tr\|Q17NG8\|Q17NG8_AEDAE | 138.5 | 16 | 13 | 13 | 96956 | Alpha-1 4 glucan phosphorylase OS=Aedes aegypti OX=7159 GN=5565921 PE=3 SV=1 |
| 12. | tr\|Q16ZI5\|Q16ZI5_AEDAE | 186.9 | 55 | 26 | 12 | 44274 | AAEL008166-PA OS=Aedes aegypti OX=7159 GN=AAEL008166 PE=4 SV=1 |
| 13. | tr\|A0A1S4FIY1\|A0A1S4FIY1_AEDAE | 186.9 | 69 | 26 | 12 | 35296 | Uncharacterized protein OS=Aedes aegypti OX=7159 GN=5570233 PE=3 SV=1 |
| 14. | tr\|J9HYM2\|J9HYM2_AEDAE | 143.81 | 45 | 11 | 11 | 35441 | Glyceraldehyde-3-phosphate dehydrogenase OS=Aedes aegypti OX=7159 GN=23687404 PE=3 SV=1 |
| 15. | tr\|Q16LP5\|Q16LP5_AEDAE | 128.13 | 24 | 11 | 11 | 56218 | Pyruvate kinase OS=Aedes aegypti OX=7159 GN=AAEL012576 PE=3 SV=1 |
| 16. | tr\|Q16F38\|Q16F38_AEDAE | 128.13 | 24 | 11 | 11 | 57457 | Pyruvate kinase OS=Aedes aegypti OX=7159 GN=5565629 PE=1 SV=1 |
| 17. | tr\|A0A1S4EXR8\|A0A1S4EXR8_AEDAE | 111.42 | 21 | 11 | 11 | 68763 | Malic enzyme OS=Aedes aegypti OX=7159 PE=3 SV=1 |
| 18. | tr\|Q17M99\|Q17M99_AEDAE | 111.42 | 21 | 11 | 11 | 72027 | Malic enzyme OS=Aedes aegypti OX=7159 GN=AAEL001091 PE=3 SV=1 |
| 19. | tr\|Q17E82\|Q17E82_AEDAE | 144.47 | 15 | 10 | 10 | 81269 | Glycerol-3-phosphate dehydrogenase OS=Aedes aegypti OX=7159 GN=5579187 PE=3 SV=1 |
| 20. | tr\|Q17E81\|Q17E81_AEDAE | 144.47 | 15 | 10 | 10 | 80970 | Glycerol-3-phosphate dehydrogenase OS=Aedes aegypti OX=7159 GN=5579187 PE=3 SV=1 |
